# Supplementary material for: Chromothripsis during telomere crisis is independent of NHEJ, and consistent with a replicative origin
Source: Genome Res. 2019 May;29(5):737–49. doi: 10.1101/gr.240705.118 (PMC6499312; doi:10.1101/gr.240705.118)
Supplement: Supplemental Material [file supp_gr.240705.118_Supplemental_file_1.zip › contigs/annotated_contigs/DB73/contig.3.DB73_length_523_mean_cov_11.4971319312.docx]

**DB73_length_523_mean_cov_11.4971319312**

```
CCAGAGAGGCTGAGGCAGGAGAATCGCTTGAACCCGGGAGGCAGAGGTTGTGGTGAGCTAAGATTGAGCCATTGCATTCTAGCCTGGGC
 >chr3:52760853-52761118 - E=5e-137 p=2e-03
AACAAGAGTGAAACTCTGTCTCAGGAAAAAAAAAAAAAAGTTTTAGATGCCTGTTAGATCTAGTTGGTTTACTGCATTGTTCAAGTCCT

CTTATTTATTTATTTGAGACGGAGTCTCGCTCTGTCACCCAGGCTGGAGTGCAATGGCGCCATCTCAGCTCACTGCAACCT|GAGTGCC

A|GCACTGCAACCTCCGCCTCCCAGGTA|CA|AGCAGTCTGGTCTTTCAGCCTTCGGACTGATTTGGCTTGAAGGTGGGGTTTCACTGG
  >chr19:3999586-3999612 - E=5e-04  >chr3:52755759-52755987 - E=1e-125
GAACCTGCCCCATCTGCCTAGGTATTTGGCCACCTCCTGTCACTATCACCAGGGATTAGTCACAGGTATAAACTTAAGGTCCTTCTCAT

GTCTGAATCTGTGTTTTCCCCTGGGCATGTACATGCATGGTAACTTTCCAAATTCCCCTGTAAATGTGGTTACTTTTTTTTT
```
